# Facile Synthesis of Polypyrrole-Decorated RGO-CuS Nanocomposite for Efficient Nickel Removal from Wastewater

**DOI:** 10.3390/polym16223138

**Published:** 2024-11-11

**Authors:** Fouzia Mashkoor, Mohd Shoeb, Mohmmad Naved Khan, Changyoon Jeong

**Affiliations:** School of Mechanical Engineering, Yeungnam University, Gyeongsan 38541, Gyeongbuk, Republic of Korea; fmashkoor@yu.ac.kr (F.M.); mshoeb@yu.ac.kr (M.S.); navedkhan@yu.ac.kr (M.N.K.)

**Keywords:** reduced graphene oxide, nickel, polypyrrole, wastewater, adsorption

## Abstract

Efficient wastewater treatment, particularly the removal of heavy metal ions, remains a challenging priority in environmental remediation. This study introduces a novel sandwich-structured nanocomposite, RGO-CuS-PPy, composed of reduced graphene oxide (RGO), copper sulfide (CuS), and polypyrrole (PPy), synthesized via a straightforward hydrothermal method. The unique combination of RGO, CuS, and PPy offers enhanced adsorption capacity for Ni(II) ions due to RGO’s high surface area and CuS’s active binding sites, supported by PPy’s structural stability contributions. This study is among the first to explore this specific nanocomposite architecture for Ni(II) removal, achieving an adsorption capacity of 166.67 mg/g and a high removal efficiency of 94.9% within 210 min for 55 mg/L of Ni(II) concentration at pH 6 and adsorbent dose of 3 mg/15 mL. The kinetic analysis shows the best fitted time-dependent experimental data with the pseudo-second-order model, indicating chemisorption. Isotherm studies confirmed the Langmuir model as the best fit, yielding a high monolayer adsorption capacity of 166.67 mg/g. Thermodynamic analysis shows the adsorption process was endothermic (ΔH° = 80.23 kJ/mol) and spontaneous (ΔG° ranging from −6.985 to −14.399 kJ/mol). Additionally, reusability tests using 0.1 M HCl for desorption demonstrated good reusability, emphasizing the RGO-CuS-PPy nanocomposite’s potential as a sustainable adsorbent for Ni(II) removal in wastewater treatment applications.

## 1. Introduction

Water is an indispensable natural resource in the 21st century, essential for sustaining human life and supporting societal development. However, the exponential growth in global population, combined with the expansion of agricultural, industrial, and urban activities, led to a significant imbalance between water supply and demand [1]. This disparity is further exacerbated by the contamination of water sources due to various pollutants, particularly toxic heavy metals released from industrial processes such as metal manufacturing, mining, and power generation [2]. Among these pollutants, nickel (Ni) is of particular concern due to its extensive use in industries such as electroplating, alloy production, and battery manufacturing, leading to its elevated presence in industrial wastewater. Nickel primarily exists in two oxidation states: Ni(0) and Ni(II), with Ni(II) being the more toxic and prevalent form in aquatic environments [3]. Ni(II) is highly soluble in water and can be readily absorbed by living organisms, leading to bioaccumulation and biomagnification within the food chain. Prolonged exposure to Ni(II) poses severe health risks, including respiratory issues, allergic reactions such as dermatitis, and even carcinogenic effects. The International Agency for Research on Cancer (IARC) classified Ni(II) compounds as Group 1 carcinogens due to their potential to induce cancer in humans [4]. Given the widespread industrial use of nickel and its associated environmental and health risks, Ni(II) is recognized as a priority pollutant by regulatory agencies such as the U.S. Environmental Protection Agency (EPA) and the European Union (EU). The World Health Organization (WHO) set a maximum permissible limit for Ni(II) in drinking water at 0.07 mg/L, underscoring the need for effective removal strategies [5].

Various techniques are utilized to eliminate harmful substances from polluted water, such as electrodialysis, chemical precipitation, photocatalysis, and membrane filtration [6]. Despite their widespread use, these traditional methods often come with notable limitations, including high energy requirements, incomplete pollutant removal, expensive machinery, and the production of sludge or solid waste that necessitates additional disposal efforts. Furthermore, they may struggle to handle large quantities of wastewater, which hampers their applicability on a larger scale. On the other hand, adsorption gained recognition as a highly efficient and environmentally friendly solution for extracting heavy metals such as nickel from water [7]. This approach is not only economical, but also features a straightforward design and operational flexibility, allowing for the regeneration of the adsorbents used [8]. The effectiveness of adsorption is significantly influenced by the properties of the adsorbent material, which is vital for achieving optimal performance [9]. Recent studies are concentrating on creating advanced adsorbents with enhanced capacities for pollutant uptake and improved regeneration for larger-scale applications. Innovations such as hybrid adsorbents, which integrate the benefits of various materials, along with functionalized nanomaterials, are being investigated to refine water treatment methods. These developments are critical for meeting the increasing need for effective, scalable, and sustainable water purification solutions.

Various nanocomposites have been extensively utilized to remove contaminants from water systems by incorporating materials such as carbon, polymers, metal oxides, and inorganic–organic composites [10]. Among these, conducting polymers such as polypyrrole (PPy) stand out due to their ease of production, cost-effectiveness, and tunable redox properties. Composed of repeating pyrrole units, PPy is recognized for its high thermal stability, excellent electrical conductivity, and chemical adaptability, making it a versatile choice for environmental remediation applications [11]. Additionally, PPy gained increased interest for its ability to protonate and facilitate ion exchange, along with long-term environmental stability, nontoxicity, and relatively low cost [12,13]. Its intrinsic conductivity facilitates improved electron transfer during adsorption, enhancing interactions with charged contaminants such as metal ions. Notably, PPy with a hierarchical structure demonstrated strong adsorption performance. Furthermore, it can form composites that effectively target both organic and inorganic pollutants in water. These composites increase the availability of functional groups for adsorption, thereby enhancing overall efficiency [14]. The incorporation of PPy-based composites can lead to more structural defects and expose additional active sites, which are beneficial for contaminant removal. However, to improve performance further, introducing functional groups such as amino (−NH_2_), hydroxyl (−OH), and sulfonic groups into the PPy backbone is essential [15].

To further enhance the properties of polypyrrole, researchers developed nanocomposites by integrating it with materials such as copper sulfide (CuS). The combination of polypyrrole with CuS presents several notable advantages for the adsorption of nickel ions (Ni(II)). CuS is recognized for its effective adsorption capabilities, particularly for heavy metals, due to its unique layered structure, which provide ample sites for interaction [16]. Additionally, CuS exhibits excellent stability and reusability, making it an ideal candidate for wastewater treatment applications. Its compatibility with polypyrrole ensures that the resulting nanocomposite maintains structural integrity even under harsh environmental conditions. The integration of CuS can improve the mechanical strength of the nanocomposite, allowing for better handling and operational efficiency. Moreover, CuS has the ability to form strong chemical bonds with Ni(II) ions, significantly enhancing the adsorption capacity. This chemical interaction not only increases the overall efficiency of Ni(II) removal, but also promotes faster adsorption kinetics, enabling quicker purification of contaminated water [16]. The incorporation of CuS can also reduce the potential for leaching, thereby minimizing the risk of secondary pollution. Furthermore, the combination of polypyrrole and CuS allows for better dispersion of the metal sulfide particles within the polymer matrix, maximizing their contact with contaminants and enhancing adsorption performance.

Additionally, anchoring the polypyrrole-CuS composite onto reduced graphene oxide (RGO) significantly boosts its functionality. RGO, characterized by its two-dimensional structure and large surface area, provides extra adsorption sites that complement the enhanced capabilities of the modified polypyrrole. The presence of oxygen-containing functional groups on RGO further facilitates interactions with metal ions, thereby improving the overall adsorption capacity and selectivity for contaminants [17]. The amalgamation of polypyrrole, CuS, and RGO results in a synergistic effect that enhances the stability, reactivity, and adsorption efficiency of the nanocomposite. This integrated material holds promise for effective and scalable solutions in wastewater purification, particularly for the removal of hazardous heavy metals such as nickel, thereby contributing to environmental remediation efforts.

The novelty of this work lies in the unique architecture of the RGO-CuS-PPy NCs, combining the high surface area of RGO, the active binding sites of CuS, and the stability-enhancing properties of polypyrrole (PPy). This study is among the first to explore this specific configuration for Ni(II) ion adsorption, demonstrating a high adsorption capacity and significant removal efficiency under optimized conditions. By evaluating the isotherm, kinetic, and thermodynamic parameters, this study provides in-depth insights into the adsorption mechanism, which has not been fully explored for this composite structure. The findings underscore the potential of RGO-CuS-PPy as a sustainable and recyclable adsorbent for heavy metal removal, contributing to advancements in wastewater treatment technology.

In this study, hydrothermally synthesized RGO-CuS-PPy was utilized to remove Ni(II) ions from aqueous solutions. The adsorption process was optimized by adjusting parameters such as the RGO-CuS-PPy dosage, pH levels, initial Ni(II) concentration, and temperature. Additionally, thermodynamic kinetic and isotherm analyses were conducted to clarify the mechanism of Ni(II) adsorption onto the RGO-CuS-PPy NCs.

## 2. Experimental

### 2.1. Material

All chemicals were obtained from Sigma-Aldrich, Seoul, Republic of Korea and used without further purification. The chemicals used include graphene oxide (GO) synthesized from graphite powder based on Hummers’ method, ethylene glycol (EG), copper(II) acetate monohydrate, thiourea, cetyltrimethylammonium bromide (CTAB), pyrrole monomer, ammonium persulfate, nickel(II) nitrate hexahydrate, ethanol, hydrochloric acid, sodium hydroxide, and distilled water. These reagents were used in synthesizing the RGO-CuS-PPy nanocomposite and in adsorption experiments.

### 2.2. Synthesis of RGO-CuS-PPy Nanocomposite

**Figure 1:**
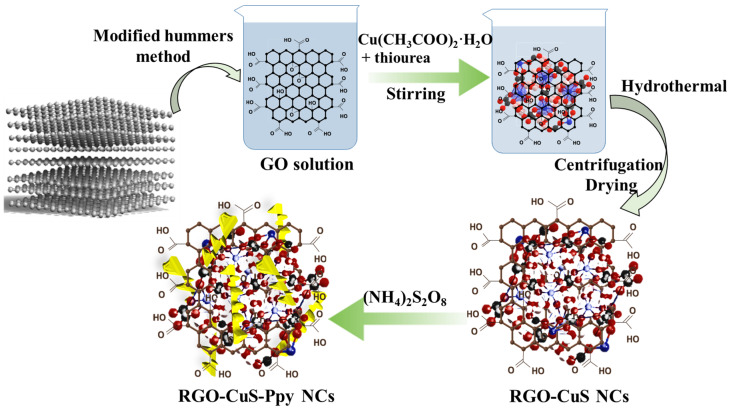
Synthesis scheme of RGO-CuS-PPy NCs.

The synthesis of RGO-CuS-PPy NCs begins with preparing RGO-CuS. Graphene oxide (GO) was prepared from graphite powders based on Hummers’ method [18]. The CuS-RGO hybrid was synthesized through a modified method based on the procedure outlined in reference [19]. In a standard procedure, 17.5 mg of graphene oxide (GO) powder was dispersed into 35 mL of ethylene glycol (EG) solution and sonicated for 1 h. Then, 99.83 mg of copper acetate (Cu(CH_3_COO)_2_·H_2_O) and 0.114 g of thiourea were gradually added to the mixture at room temperature. After stirring vigorously for about 30 min, the mixture was transferred into a Teflon-lined stainless steel autoclave and heated at 180 °C for 12 h. The solution was then allowed to cool to room temperature naturally. To remove any unreacted materials, the resulting product was washed multiple times with ethanol. Finally, the CuS-RGO hybrid was collected by centrifugation and dried under a vacuum at 50 °C overnight. A specific amount of the synthesized CuS-RGO nanocomposites (NCs) was then added to a 1 M solution of the CTAB surfactant prepared in a 1:1 ethanol and water mixture, followed by stirring for 1 h at room temperature. Pyrrole monomer was then introduced into the mixture and thoroughly stirred. After 1 h, 1.15 mM ammonium persulfate was gradually added to initiate polymerization, which proceeded with continuous stirring for 24 h at room temperature (Figure 1). The resulting grey-black precipitate was filtered and washed step by step with ethanol, dichloromethane, and deionized water to remove impurities such as oligomers, monomers, and unreacted oxidants. The final product was RGO-CuS-Ppy NCs (Figure 1). Detailed procedures for Ni(II) adsorption experiments can be found in the Supplementary Materials.

### 2.3. Batch Adsorption Studies

The Ni(II) ion adsorption experiments using RGO-CuS-PPy NCs as adsorbents were conducted as follows: To determine the effect of adsorbent dosage, various amounts of RGO-CuS-PPy NCs (ranging from 1 to 30 mg per 15 mL of Ni(II) solution) were used. The adsorption process was monitored over time, with sampling intervals from 2 to 240 min. The effect of pH on adsorption was investigated by adjusting the solution’s pH between 2 and 8 using 0.1 M NaOH or 1 M HCl. Additionally, experiments were conducted across a temperature range of 298 to 323 K to evaluate the temperature’s impact on adsorption efficiency. For each experiment, the solution in the flask was mixed on a reciprocal shaker. At pre-set intervals, a certain amount of the suspension was withdrawn from the system by centrifugation. The supernatant from each interval was analyzed using inductively coupled plasma atomic emission spectroscopy (ICP-AES; OPTIMA 8300, Perkin Elmer, Seoul, Republic of Korea) to measure the residual Ni(II) concentration. The amount of Ni(II) adsorbed onto the RGO-CuS-PPy NCs at any time or at equilibrium (q_t_/q_e_) was calculated based on the concentration change of Ni(II) in the solution, using the following equation (Equation (1)):

(1) $$q_{t/e}=\left(C_{o}-C_{t/e}\right)\times \frac{V}{m}.$$

The removal efficiency (%R) was obtained by using the following expression (Equation (2)):

(2) $$\%R=\frac{\left(C_{o}-C_{t/e}\right)}{C_{o}}\times 100$$

where C_o_/C_t_/C_e_ (mg/L) signifies the initial/any time/equilibrium liquid-phase concentrations of solute, respectively. “V” and “m” are the volume in ml of the Ni(II) solution and mass in g of the RGO-CuS-PPy NCs, respectively.

The linear forms of the psuedo-first-order, pseudo-second-order, and intraparticle diffusion models are described in Equations (3)–(5) [20,21]. In these equations, *K*_1_, *K*_2_, and *K*_3_ are the equilibrium rate constants of the psuedo-first-order, pseudo-second-order, and intraparticle diffusion models, respectively, while *q_fo_* and *q_so_* represent the equilibrium adsorption capacities (mg/g) of the psuedo-first-order and pseudo-second-order models, respectively. *C* is a constant.

P-fo:

(3) $${ln(q}_{fo}-q_{t})={lnq}_{f_{o}}-K_{1}t$$

P-so:

(4) $$\frac{t}{q_{t}}=\frac{1}{K_{2}q_{so}^{2}}+\frac{t}{q_{so}}$$

I-D:

(5) $$q_{t}=K_{3}t^{0.5}+C$$

The experimental data were fitted using two adsorption isotherms models, namely, Langmuir and Freundlich models to theoretically explore the adsorption process. The isotherm models are expressed in Equations (6) and (7). All adsorption isotherm models were used by the nonlinear form of *q_e_* versus *C_e_*. Here, *q_L_*, is a maximum adsorption capacity (mg/g) of Langmuir. Meanwhile, *K_L_* (L/mol) and *K_F_* (L/mg), respectively, are the constants of Langmuir and Freundlich models, which are related to the adsorption capacity or the interaction between the adsorbent and the adsorbate. Furthermore, “n” serves as a constant in the Freundlich model, indicating the strength of adsorption. Here, R (8.314 J/mol/K) is the universal gas constant, and T represents the temperature measured in Kelvin.

(6) $$q_{e}=\frac{q_{L}K_{L}C_{e}}{1+K_{L}C_{e}}$$

(7) $$q_{e}=K_{F}C_{e}^{\frac{1}{n}}$$

In these isotherms, the Langmuir model (Equation (6)) and the empirical Freundlich formula (Equation (7)) are the monolayer sorption on the uniform surface and multilayer sorption on the heterogeneous surface, respectively.

The thermodynamic parameters: enthalpy (∆H), Gibbs free energy (∆G), and entropy (∆S), were determined based on calculations from Equations (8) and (9).

(8) $$∆G=-RTlnK_{c}$$

(9) $${lnK}_{c}=\frac{-∆G}{RT}=\frac{∆S}{R}-\frac{∆H}{RT}$$

where K*_c_* is the thermodynamic constant and can be calculated by the ratio of adsorption capacity to the residual concentration of metal ion molecule in the solution.

### 2.4. Instrumental Characterization

The adsorbent was examined by FTIR, SEM, TEM, and XRD techniques. The FTIR spectra from 4000 to 400 cm^−1^ were recorded for the analyses of functional groups using Nicolet IS50. Infrared analysis was conducted using an FTIR spectrometer from Thermo Fisher Scientific, Seoul, Republic of KoreaKorea. To investigate surface morphology, we utilized both scanning electron microscopy (SEM, JEOL JSM6510LV, Tokyo, Japan) and transmission electron microscopy (TEM, JEOL 2100, Tokyo, Japan). The composite’s crystallinity was examined by X-ray powder diffraction (XRD) on a MiniFlex™ II benchtop system (Rigaku Corporation, Tokyo, Japan), with measurements performed over a 2θ range of 10–80°, at 40 kV and 30 mA.

## 3. Results and Discussion

**Figure 2:**
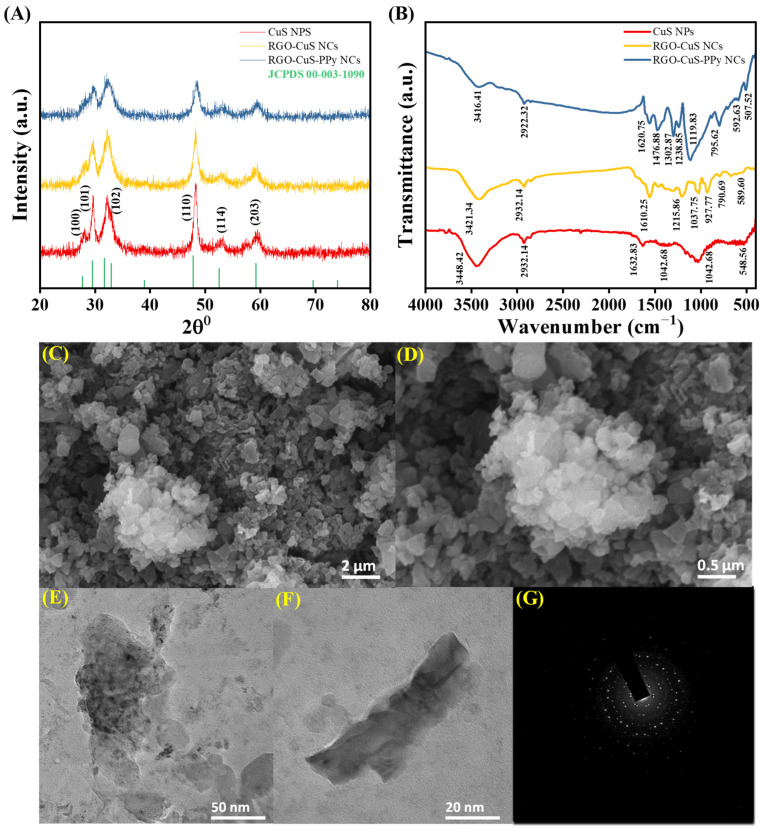
(**A**) XRD (**B**) FTIR of CuS, CuS-PPy, and RGO-CuS-PPy, (**C**,**D**) SEM, (**E**,**F**) TEM, and (**G**) SAED of RGO-CuS-PPy NCs.

The X-ray diffraction (XRD) patterns provide information on the crystal structure of CuS NPs, RGO-CuS NCs, and RGO-CuS-PPy NCs as shown in Figure 2A. The peaks in the CuS NPs are matched to the (102), (103), (006), and (110) planes of hexagonal CuS based on the JCPDS standard 00-003-1090 [22]. These sharp, well-defined peaks indicate high crystallinity. For the RGO-CuS NCs, the peaks appear at similar 2θ values, confirming that the hexagonal structure of CuS was retained. However, the peaks are slightly broader, which suggests smaller crystallite sizes or lattice strain caused by the interaction between CuS and RGO. This broadening may also result from the dispersion of CuS on RGO, leading to smaller crystal domains or defects. In the RGO-CuS-PPyNCs, the peaks broaden further, and their intensity decreases. This points to a significant reduction in crystallinity, likely due to the addition of polypyrrole, which introduces amorphous regions and reduces the crystallite size. The broadening of the (102) and (103) peaks suggests that PPy disrupts crystal growth along these planes. Despite this, no new peaks are observed, indicating that PPy does not form detectable crystalline phases due to the high abundance of CuS NPs crystallinity. The core CuS structure remains intact across all samples, but adding RGO and PPy reduces crystallite size and increases disorder. This structural change could enhance the surface properties of the composites, making them suitable for adsorption applications.

The FTIR spectra of CuS NPs, RGO-CuS NCs, and RGO-CuS-PPy NCs reveal essential structural and chemical insights (Figure 2B). In the spectrum of CuS NPs, characteristic peaks around 600–500 cm^−1^ are attributed to Cu–S stretching vibrations, confirming the formation of copper sulphide [23]. In the RGO-CuS nanocomposite, the Cu–S bands are preserved, indicating the retention of the CuS structure. Additionally, a broad peak around 3421 cm^−1^ corresponds to O–H stretching vibrations, likely from hydroxyl groups on RGO [24]. In comparison, peaks near 1610 cm^−1^ are associated with C=C stretching of the sp^2^ hybridized carbon network in RGO, confirming its presence [25]. These oxygen-containing functional groups play a crucial role in enhancing the stability and dispersion of CuS on RGO sheets, potentially improving the material’s overall properties. In the FTIR spectrum of the RGO-CuS-PPy nanocomposite, the broad peak around 3416 cm^−1^ is attributed to overlapping O-H and N-H stretching vibrations, indicating the presence of hydroxyl groups and nitrogen-containing functionalities [26]. Notable bands at 1476 cm^−1^ and 1620 cm^−1^ correspond to pyrrole ring stretching vibrations, alongside C=C stretching within the sp^2^ carbon network of RGO. Additional peaks observed at 1302 cm^−1^ (C–N stretching) and the peak in between 1200 cm^−1^ and 1000 cm^−1^ are due to the aromatic ring bending confirming the successful incorporation of polypyrrole into the nanocomposite [27]. Notably, the Cu–S peaks remain present in all composites, indicating that the CuS structure is retained even after integrating RGO and PPy, ensuring that the CuS maintains its chemical reactivity and stability. The combination of RGO, CuS, and PPy in the composite materials provides a synergistic effect. While CuS offers chemical activity and structural integrity, RGO enhances the composite’s surface area and electrical conductivity, and PPy adds flexibility and further improves conductivity. The FTIR spectra, therefore, confirm that integrating RGO and PPy into the CuS matrix improves the overall functionality of the material without compromising the core structure of CuS.

The SEM images of the RGO-CuS-PPy NCs provide essential details about its morphology at two magnifications: 2 µm (Figure 2C) and 0.5 µm (Figure 2D). At the 2 µm scale, the surface looks rough and porous, indicating a high surface area that is beneficial for adsorption. The structure shows agglomerated particles, with white clusters likely representing aggregated CuS nanoparticles embedded within the RGO and PPy matrix. This porous structure enhances the available surface for adsorption. At the 0.5 µm scale, the individual particles become more apparent, revealing smaller, densely packed crystalline structures. The bright white areas correspond to well-dispersed CuS nanoparticles, though some aggregation is still visible. The RGO sheets, while less distinct due to their thin nature, provide essential support that integrates with the CuS nanoparticles and PPy chains. The overall granular and interconnected texture suggests that these components are successfully integrated. Key takeaways from the SEM images include the presence of particle aggregation, which might reduce the surface area of individual nanoparticles but contribute to a highly porous structure. This porosity is crucial for enhancing interactions with target molecules, making the composite effective for adsorption applications. Additionally, the rough and granular texture indicates good dispersion of CuS nanoparticles within the RGO and PPy matrix, highlighting that RGO offers conductive support, while PPy improves flexibility and conductivity. Overall, the RGO-CuS-PPy composite displays a morphology that is well-suited for various high-performance applications.

The TEM image shown in Figure 2E,F of the RGO-CuS-PPy NCs highlights the sample’s morphology and crystalline characteristics at different scales. Figure 2E, with a scale bar of 50 nm, shows the overall morphology of the composite. In this image, the darker regions likely represent CuS NPs, which exhibit higher electron density due to their metallic nature, making them appear more distinct than the surrounding matrix. The lighter, more transparent regions are probably RGO sheets. These sheets act as a matrix, providing a large surface area for the distribution of CuS NPs and polypyrrole, a conductive polymer likely present as a thin coating on the surface of the RGO or CuS particles. This multi-phase composite suggests a well-distributed mixture of metal sulfide, polymer, and graphene components, potentially leading to enhanced electrical conductivity and catalytic properties. Figure 2F, with a scale bar of 20 nm, offers a higher magnification view, focusing on a smaller composite section. Here, the elongated, darker structure represents individual CuS NPs with PPy or aggregation, providing a clearer view of the nanostructures in the material. The higher resolution reveals the fine details of the nanocomposite, confirming the nanoscale dimensions of the CuS particles embedded within the RGO matrix. This image highlights the intimate interaction between the components, critical for applications requiring high surface area and efficient charge or ion transport. Figure 2G, the selected area electron diffraction pattern (SAED), further elucidates the crystallographic nature of the composite. The pattern shows distinct diffraction spots arranged in concentric rings, a hallmark of a polycrystalline material. These rings indicate the crystalline phases within the sample, likely arising from the CuS nanoparticles. The multiple rings correspond to various crystal planes of the CuS phase, confirming its crystalline nature. In contrast, RGO and PPy are likely amorphous or weakly crystalline, contributing less to the diffraction pattern. The presence of sharp diffraction spots along these rings further confirms the excellent crystallinity of the CuS nanoparticles. The TEM images and SAED pattern collectively provide detailed insight into the RGO-CuS-PPy composite, demonstrating the well-dispersed nature of CuS nanoparticles within the RGO and PPy matrix, as well as the crystalline structure of the CuS component. Figure S1 shows that the BET-specific surface area for the RGO-CuS-PPy NCs was found to be 83.67 m^2^/g with an average pore diameter of 23.54 nm.

### Adsorption Study

**Figure 3:**
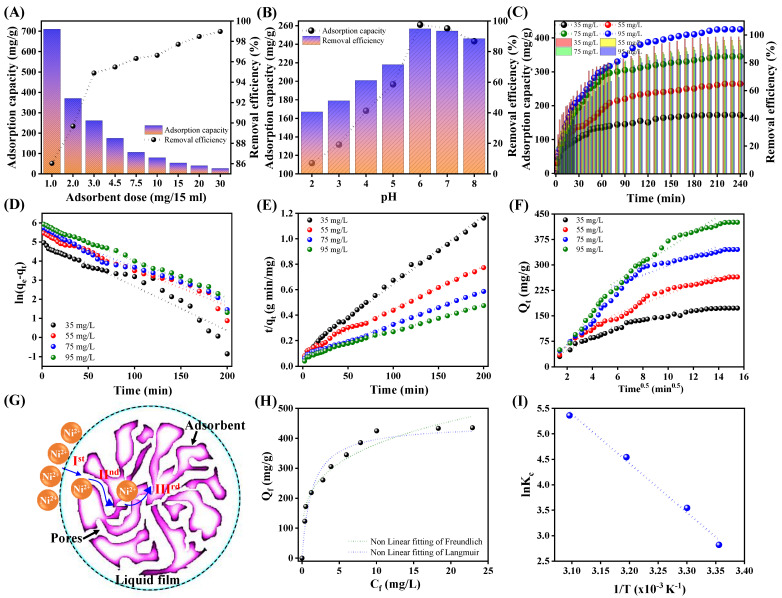
Effects on Ni(II) adsorption using RGO-CuS-PPy NCs: (**A**) variation with nanocomposite dose; (**B**) influence of pH; (**C**) Impact of interaction time (bar denoted removal efficiency and black dotted line with circle shows adsorption capacity); linear kinetics graphs: (**D**) pseudo-first order (**E**) pseudo-second order; (**F**,**G**) IPD model and pictorial representation of Ni(II) diffusion into the RGO-CuS-PPy NCs (Ist (surface diffusion), IInd (pore diffusion), and IIIrd (equilibrium approaching stage); (**H**) non-linear plots of Freundlich and Langmuir models, and (**I**) lnKc vs. 1/T plot for the sorption of Ni(II) onto the RGO-CuS-PPy.

A set of experiments was carried out to investigate the influence of varying dosages of RGO-CuS-PPy NCs on the adsorption performance for Ni(II) ions of 55 mg/L of concentration. Adsorbent amounts ranged from 1 mg/15 mL to 30 mg/15 mL, as depicted in Figure 3A. The findings indicate that increasing the adsorbent dosage led to a substantial rise in removal efficiency, while the adsorption capacity per unit mass declined. For instance, when the dosage was increased from 1 mg/15 mL to 3 mg/15 mL, the removal efficiency improved from 86.04% to 94.9%, but the adsorption capacity decreased from 709.8 mg/g to 260.98 mg/g. This reduction in capacity can be attributed to the lower concentration gradient between the adsorbent and Ni(II) ions, which results in fewer ions being adsorbed per unit mass. The enhanced removal efficiency with higher adsorbent dosages can be explained by the increased availability of active sites and functional groups, which facilitate the capture of more Ni(II) ions. However, beyond 3 mg/15 mL, further increases in adsorbent dosage had minimal impact on removal efficiency, suggesting that the concentration of Ni(II) became the limiting factor in the system. This indicates that 3 mg/15 mL is the optimal adsorbent dosage for achieving the best balance between high removal efficiency and adsorption capacity, as increasing the dosage beyond this point offers no significant improvement in performance. Thus, this dosage is considered the most effective for maximizing Ni(II) ion removal.

**Scheme 1:**
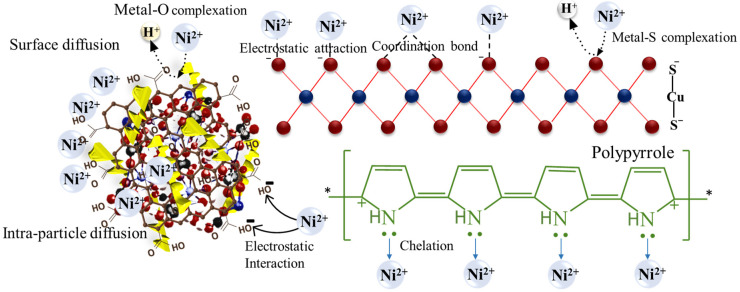
The adsorption mechanism of Ni(II) onto the RGO-CuS-PPy NCs (“*” denoted as polymeric chain).

The pH of the solution is a key factor in the adsorption of Ni(II) onto RGO-CuS-PPy nanocomposites, as it affects both the chemical state of the Ni(II) ions and the surface charge of the adsorbent. In this study, the adsorption of Ni(II) was optimized under conditions of a 210 min contact time, an initial Ni(II) concentration of 55 mg/L, and an adsorbent dosage of 3 mg per 15 mL (Figure 3B). After evaluating the effect of pH on the adsorption process, pH 6 was determined to be the optimal condition for achieving maximum removal efficiency. At lower pH levels, the surface functional groups of the RGO-CuS-PPy NCs, such as −NH_2_ and −OH, become highly protonated. This leads to significant electrostatic repulsion between the positively charged surface and the Ni(II) ions, reducing the efficiency of adsorption. As the pH increases, the extent of protonation decreases, weakening the electrostatic repulsion and allowing stronger interactions between Ni(II) and the surface functional groups. This results in enhanced adsorption capacity up to pH 6, where optimal adsorption was achieved. Beyond pH 6, however, the adsorption efficiency starts to decline due to the increasing competition from hydroxide ions, which can form nickel hydroxide species. These species precipitate out of solution, reducing the availability of free Ni(II) ions for adsorption and interfering with the adsorption process. In conclusion, pH 6 was identified as the optimal pH for the adsorption of Ni(II) onto RGO-CuS-PPy NCs, balancing the need to minimize electrostatic repulsion while avoiding the formation of competing nickel hydroxide species. Scheme 1 shows the adsorption of Ni(II) onto RGO-CuS-PPy NCs at pH 6 is driven by a combination of electrostatic attraction, ion exchange, and complexation mechanisms. These interactions enable efficient binding of Ni(II) to the functional groups of RGO-CuS-PPy NCs, resulting in high adsorption capacity.

To evaluate the equilibrium time for the adsorption of Ni(II) ions onto RGO-CuS-PPy NCs, a series of experiments were conducted at varying initial concentrations of Ni(II) (35 mg/L, 55 mg/L, 75 mg/L, and 95 mg/L). The time-dependent adsorption experiment was assessed over a duration of 240 min. As illustrated in Figure 3C, the adsorption of Ni(II) exhibited a rapid increase during the initial phase of the experiment, which can be attributed to the abundant active sites available on the surface of the adsorbent. This facilitated the swift capture of Ni(II) ions from the solution. However, as the adsorption process continued, the rate of uptake gradually slowed, ultimately reaching equilibrium at approximately 210 min. This deceleration can be explained by the diminishing number of available active sites, leading to increased repulsion between the adsorbed Ni(II) ions and the free ions still present in the solution. The equilibrium adsorption capacity demonstrated a notable increase with Ni(II) concentration, ranging from 172.49 mg/g at 35 mg/L to 424.88 mg/g at 95 mg/L. This trend indicates that higher initial concentrations of Ni(II) significantly enhance the adsorption capacity of the RGO-CuS-PPy NCs. Conversely, the removal efficiency exhibited a decrease from 98.56% at 35 mg/L to 89.45% at 95 mg/L. This reduction in efficiency can be attributed to the saturation of available active sites and the competition between adsorbed and free Ni(II) ions as the concentration in the solution rises. Furthermore, the observed increase in adsorption capacity can be explained by several interrelated factors. As the initial concentration of Ni(II) rises, the greater availability of metal ions in the solution creates a stronger driving force for adsorption, enhancing the likelihood of Ni(II) ions adhering to the adsorbent’s active sites. This process is also facilitated by improved mass transfer dynamics at higher concentrations. However, the decline in removal efficiency may be due to the rapid saturation of active sites, leading to fewer available sites for additional adsorption. Moreover, increased competition for these limited sites intensifies repulsive interactions between adsorbed and free Ni(II) ions, further inhibiting the adsorption process [28].

To further examine the adsorption of Ni(II) onto RGO-CuS-PPy, the time-dependent adsorption data were applied on the three well-known kinetic models: pseudo-first-order, pseudo-second-order, and intraparticle diffusion models. The pseudo-first-order model indicates that the adsorption process is primarily physical, with the rate largely dependent on the diffusion of Ni(II) molecules. In contrast, the pseudo-second-order points to a significant role for chemisorption, characterized by electron sharing or van der Waals interactions between Ni(II) and the RGO-CuS-PPy material [29]. As illustrated in Figure 3D,E and Table 1, the fitting results of the pseudo-second-order yielded high correlation coefficients as compared to the pseudo-first-order, suggesting that chemical adsorption is the predominant mechanism in the interaction between RGO-CuS-PPy and Ni(II). The analysis of the intraparticle diffusion model, presented in Figure 3F,G, reveals three distinct phases in the adsorption process: (I) Ni(II) ions diffuse onto the surface of the adsorbent; (II) the ions then infiltrate the interior of the material through its porous structure; (III) finally, equilibrium is reached, leading to the saturation of the adsorption sites. The results for the intraparticle diffusion model indicate that the initial step (k_i1_) has the highest adsorption rate, which gradually decreases in the subsequent stage (k_i2_) until equilibrium is achieved (k_i3_). This pattern highlights that the adsorption rate is most rapid at the outset and slows down as the process continues.

In this study, both the Langmuir and Freundlich models were applied to analyze the interactions between the pollutant and the adsorbent. The fitting curves and related parameters are shown in Figure 3H and Table 2. For Ni(II), the Langmuir model exhibited a higher R^2^ value compared to the Freundlich model, suggesting that the adsorption of Ni(II) is better described by the Langmuir model and follows a monolayer adsorption mechanism. The maximum adsorption capacity of RGO-CuS-PPy NCs for Ni(II), based on the Langmuir model, was calculated to be 140.85 mg/g. Additionally, the separation factor ($R_{L}=\frac{1}{1+K_{L}C_{o}}$) was determined using the Langmuir model to further assess the feasibility of the adsorption process. The value of *R_L_* indicates the adsorption behavior: *R_L_* > 1: unfavorable; *R_L_* = 1: linear; 0 < *R_L_* < 1: favorable; and *R_L_* = 0: irreversible. The adsorption of Ni(II) onto the RGO-CuS-PPy NCs was found to be less than 1 at all concentrations, indicating that the adsorption process was favorable. A comparison of the adsorption performance between RGO-CuS-PPy NCs and other adsorbents [30,31,32,33,34,35] is shown in Table 3, where it is evident that the RGO-CuS-PPy NCs for the adsorption of Ni(II) presented the comparable results as that of the previously reported adsorbents. Therefore, the synthesized RGO-NiFe_2_O_4_-SiO_2_-Pln NCs can be considered a highly effective adsorbent for the treatment of wastewater.

The impact of temperature on the elimination of Ni(II) using RGO-CuS-PPy NCs was carefully examined, as illustrated in Figure S2 and detailed in Table 4. When the temperature was increased from 298 K to 323 K, the efficiency of Ni(II) removal improved significantly, rising from 894.37% to 99.53%. This trend indicates that higher temperatures facilitate the adsorption process, likely due to enhanced kinetic energy that promotes the interaction between the Ni(II) ions and the adsorbent. Additionally, thermodynamic parameters such as Gibbs free energy (ΔG), enthalpy change (ΔH), and entropy change (ΔS) calculated from the slope and intercept of lnK_c_ vs. 1/T plot (Figure 3I) are summarized in Table 4. The findings reveal that ΔG values were negative and decreased from −6.985 to −14.399 kJ/mol with increasing temperature, suggesting that the adsorption of Ni(II) is a spontaneous process [36]. The positive values of ΔH and ΔS imply that the adsorption is endothermic and associated with an increase in entropy [37].

**Figure 4:**
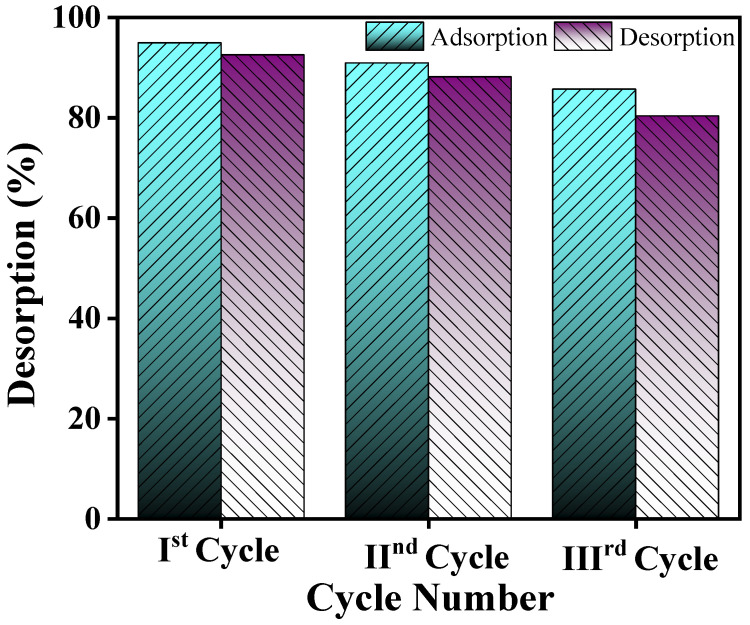
Adsorption and desorption performance in the cycling experiment.

Desorption experiments were performed by agitating Ni(II)-loaded RGO-CuS-PPy NCs NCs with 0.1 M HCl, 0.1 M NaOH, C_2_H_5_OH, and distilled water and equilibrates for 3 h and then centrifuging each sample. After centrifugation, the supernatant was analyzed and percent desorption (%*D*) was computed by employing the following relation [38]:

(10) $$\%D=\frac{m_{d}}{m_{a}}\times 100$$

where *m_a_* and *m_d_* represent the concentrations of Ni(II) ion adsorbed and desorbed in mg/L, respectively. The results show that the highest desorption was observed with the 0.1 M HCl (Table 5) and desorption efficiency remained above 80.3% for up to three adsorption–desorption cycles and the adsorption efficiency of the nanocomposite decreased by 14% after the third cycle, indicating good reusability over multiple cycles (Figure 4).

## 4. Conclusions

The present study demonstrates the effective utilization of the RGO-CuS-PPy NCs synthesized via hydrothermal process as adsorbent for the removal of Ni(II) from aqueous solutions. The adsorption efficiency of the RGO-CuS-PPy NCs was influenced by several critical parameters, including the adsorbent dosage, contact time, pH of the solution, initial Ni(II) concentration, and temperature of the system. The synthesized RGO-CuS-PPy exhibited excellent adsorption properties, achieving a removal efficiency of around 94.90% at an initial Ni(II) concentration of 55 mg/L, solution pH of 6, and in an equilibrium time of 210 min. The Langmuir isotherm model suggests that Ni(II) ions form a monolayer on the surface of the RGO-CuS-PPy NCs, with a maximum adsorption capacity of 448.46 mg/g. Time-dependent experimental data were found to be best fitted with the pseudo-second-order kinetic model. Overall, the study concludes that the RGO-CuS-PPy NCs is a highly effective adsorbent with excellent adsorption properties and high capacity. Its efficient removal of Ni(II) ions from contaminated water sources, along with its stability under varying conditions, makes it a promising candidate for large-scale wastewater treatment and environmental remediation applications.

## Figures and Tables

**Table 1:** Kinetic parameters for removal of Ni(II) onto RGO-CuS-PPy NCs (experimental parameters: RGO-CuS-PPy NCs dose = 3 mg/15 mL, temperature = 303 K, pH = 6).

Kinetics	Parameters				
Experimental	Co (mg/L)	35	55	75	95
	q_exp_ (mg/L)	172.485	260.975	345.175	424.87
Pseudo-first-order	q_fo_ (mg/g)	147.24	211.410	253.027	383.369
	K_1_ (g/mg min)	0.0232	0.0189	0.0184	0.0194
	R^2^	0.9358	0.9821	0.9719	0.9825
Pseudo-second-order	q_so_ (mg/g)	185.185	277.778	370.370	454.545
	K_2_ (g/mg min)	2.748 × 10^−4^	1.3846 × 10^−4^	1.11298 × 10^−4^	7.086 × 10^−5^
	R^2^	0.9957	0.9913	0.9966	0.9960
Intra-particle diffusion model	K_i,1_ (mg/g min^−1/2^)	17.878	25.533	36.938	46.761
	R^2^	0.947	0.968	0.9779	0.9879
	K_i,2_ (mg/g min^−1/2^)	7.330	14.441	14.289	23.668
	R^2^	0.9488	0.9219	0.8467	0.9527
	K_i,3_ (mg/g min^−1/2^)	0.267	5.3917	2.8012	2.768
	R^2^	0.6217	0.8063	0.5704	0.6281

**Table 2:** Isotherm model factors for the elimination of Ni(II) onto RGO-CuS-PPy NCs (experimental parameters: dose = 3 mg/15 mL, temperature = 303 K, pH = 6).

Isotherms	Parameters	Ni(II)
Langmuir	q_L_ (mg/g)	448.46
	K_L_ (L/g)	0.7589
	R^2^	0.9649
Freundlich	K_F_ (mg/g)	207.819
	n_F_ (L/g)	0.2629
	R^2^	0.9403

**Table 3:** Comparison with the reported literature data for the adsorption of Ni(II) onto the various adsorbents.

Adsorbent	Equilibrium Time (min)	pH	q_m_ (mg/g)	References
Ppy/clinoptilolite	24 h	4	172.00	[30]
Fe_3_O_4_/MoS_2_/chitosan	15	5	19.00	[31]
Ppy/Fe_3_O_4_	150	6	19.920	[32]
Fe_3_O_4_/SiO_2_/PPy	-	6.4	98.00	[33]
WO_3_–WS_2_/PPy	150	6	227.270	[34]
PPy/MWCNT	60	6	409.4	[35]
Fe_3_O_4_/MoS_2_/chitosan	15	5	19.00	[31]
RGO-CuS-PPy NCs	210	6	448.46	Our work

**Table 4:** Thermodynamic parameters for the elimination of Ni(II) onto RGO-CuS-PPy (experimental conditions: RGO-CuS-PPy NCs dose = 3 mg/15 mL, pH = 6, and concentration of Ni(II) = 55 mg/L).

T (K)	∆S° (J/K/mol)	∆H° (kJ/mol)	∆G° (kJ/mol)	R^2^
298	293.500	80.231	−6.985	0.9927
303			−8.931	
313			−11.819	
323			−14.399	

**Table 5:** Percentage desorption heavy metal ions from RGO-CuS-PPy NCs.

Desorbing Agent	Percentage Desorption (%D)
0.1 M HCl	92.52
0.1 M NaOH	35.08
C_2_H_5_OH	67.34
Distilled water	20.67

## Data Availability

The original contributions presented in the study are included in the article, further inquiries can be directed to the corresponding author/s.

## Supplementary Materials

The following supporting information can be downloaded at: https://www.mdpi.com/article/10.3390/polym16223138/s1, Figure S1: N_2_ isotherm adsorption–desorption analysis for RGO-CuS-PPy NCs (inset pore distribution analysis); Figure S2: Effect of temperature on the removal efficiency of Ni(II) onto the RGO-CuS-PPy NCs.
