# Supplementary material for: Facile Synthesis of Polypyrrole-Decorated RGO-CuS Nanocomposite for Efficient Nickel Removal from Wastewater
Source: Polymers (Basel). 2024 Nov 11;16(22):3138. doi: 10.3390/polym16223138 (PMC11598025; doi:10.3390/polym16223138)
Supplement: Supplementary file 1 [file polymers-16-03138-s001.zip › polymers-3277695-supplementary.pdf]

Facile Synthesis of Polypyrrole-Decorated RGO-CuS Nanocomposite for Efficient Nickel Removal fromWastewater

Fouzia Mashkoor †, Mohd Shoeb †, Mohmmad Naved Khan and Changyoon Jeong *
School of Mechanical Engineering, Yeungnam University, Gyeongsan 38541, Gyeongbuk, Republic of Korea; fmashkoor@yu.ac.kr (F.M.); mshoeb@yu.ac.kr (M.S.); navedkhan@yu.ac.kr (M.N.K.)
* Correspondence: yoonni22@yu.ac.kr
† These authors contributed equally to this work.

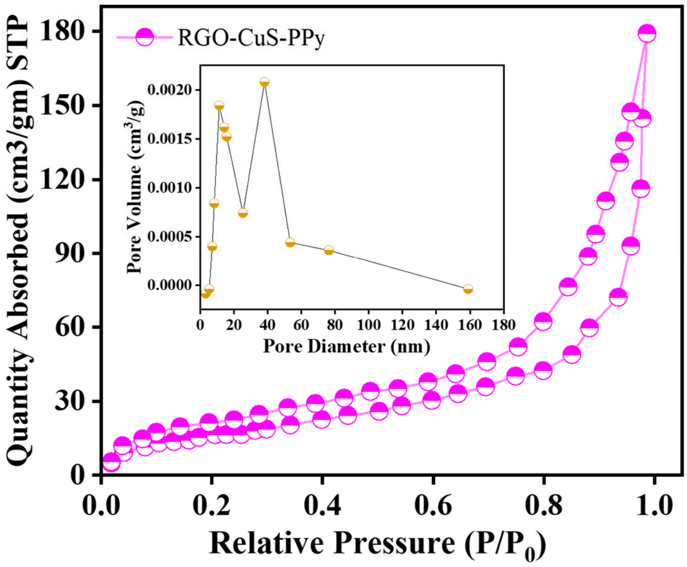

Figure S1. N_2 isotherm adsorption-desorption analysis for RGO-CuS-PPy NCs (inset pore distribution analysis).

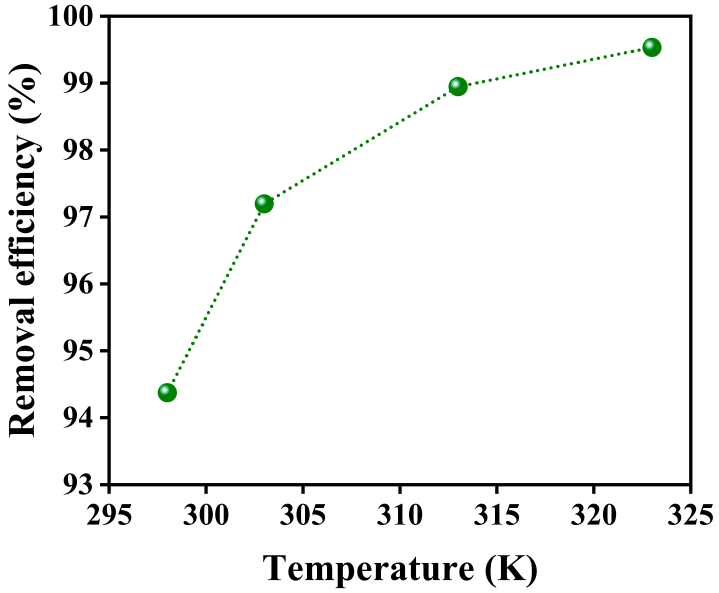

Figure S2. Effect of temperature on the removal efficiency of Ni(II) onto the RGO-CuS-PPy NCs
